# Pulmonary coinfection of *Mycobacterium tuberculosis* and *Tropheryma whipplei*: a case report

**DOI:** 10.1186/s13256-021-02899-y

**Published:** 2021-07-09

**Authors:** Binghua Zhu, Jing Tang, Rong Fang, Xuejie Fei, Qing Wang, Wenqing Wang, Xueqin Wu, Chao Liu, Qian Wang

**Affiliations:** 1 Shuguang Hospital Affiliated to Shanghai University of Traditional Chinese Medicine, Shanghai, 200021 China; 2 Shanghai Guanghua Hosptial of Integrated Traditional Chinese and Western Medicine, Shanghai, 200052 China; 3 Hangzhou Matridx Biotechnology Co., Ltd, Bd 5, 208 Zhenzhong Road, Hangzhou, 311113 Zhejiang China

**Keywords:** *Mycobacterium tuberculosis*, *Tropheryma whipplei*, Pulmonary infection, Metagenomic next-generation sequencing

## Abstract

**Background:**

We diagnosed a clinical case of pulmonary infection involving *Mycobacterium tuberculosis* and *Tropheryma whipplei* in a patient with acute respiratory distress syndrome. The diagnosis was assisted by metagenomic next-generation sequencing of bronchoalveolar lavage fluid.

**Case presentation:**

A 44-year-old Han Chinese inmate was transferred to the emergency department because of dry cough, chest tightness, and shortness of breath. The patient’s body temperature rose to 39.3 °C following empirical cephalosporin treatment for 1 week. The blood CD4+/CD8+ ratio was 0.7, suggesting immunodeficiency. Routine microbiological tests were performed, and tuberculosis interferon gamma release assays were positive. *Mycobacterium tuberculosis* polymerase chain reaction was also positive. Chest computed tomography scan revealed miliary nodules and ground-glass opacifications, which were in accordance with tuberculosis. To fully examine the etiology, we performed routine laboratory tests and metagenomic sequencing, the results of which indicated the presence of *Mycobacterium tuberculosis* and *Tropheryma whipplei.* We administered anti-tuberculosis regimen in combination with trimethoprim/sulfamethoxazole. The patient recovered, with chest computed tomography scan showing absorption of lesions.

**Conclusions:**

Compared with traditional diagnostic methods such as culture and serology, metagenomic next-generation sequencing has the advantage of detecting a wide array of microorganisms in a single test and therefore can be used for clinical diagnosis of rare pathogens and microbial coinfections. It is particularly useful for immunocompromised patients as they are more prone to infection by opportunistic microorganisms.

## Background

Here we review a case of community-acquired pneumonia caused by *Mycobacterium tuberculosis* (MTB) and *Tropheryma whipplei* (TW). MTB is a species of pathogenic bacteria in the family *Mycobacteriaceae* and the causative agent of tuberculosis [1]. TW is a Gram-positive *Actinobacteria* and the causative agent of Whipple’s disease [2]. TW is commonly found in the environment and can be carried by 1.5–7% of healthy individuals without causing symptoms [3]. MTB may occur concomitantly with other infections such as human immunodeficiency virus (HIV) [4]. However, clinical reports of MTB and TW coinfection are scarce.

## Case presentation

**Fig. 1:**
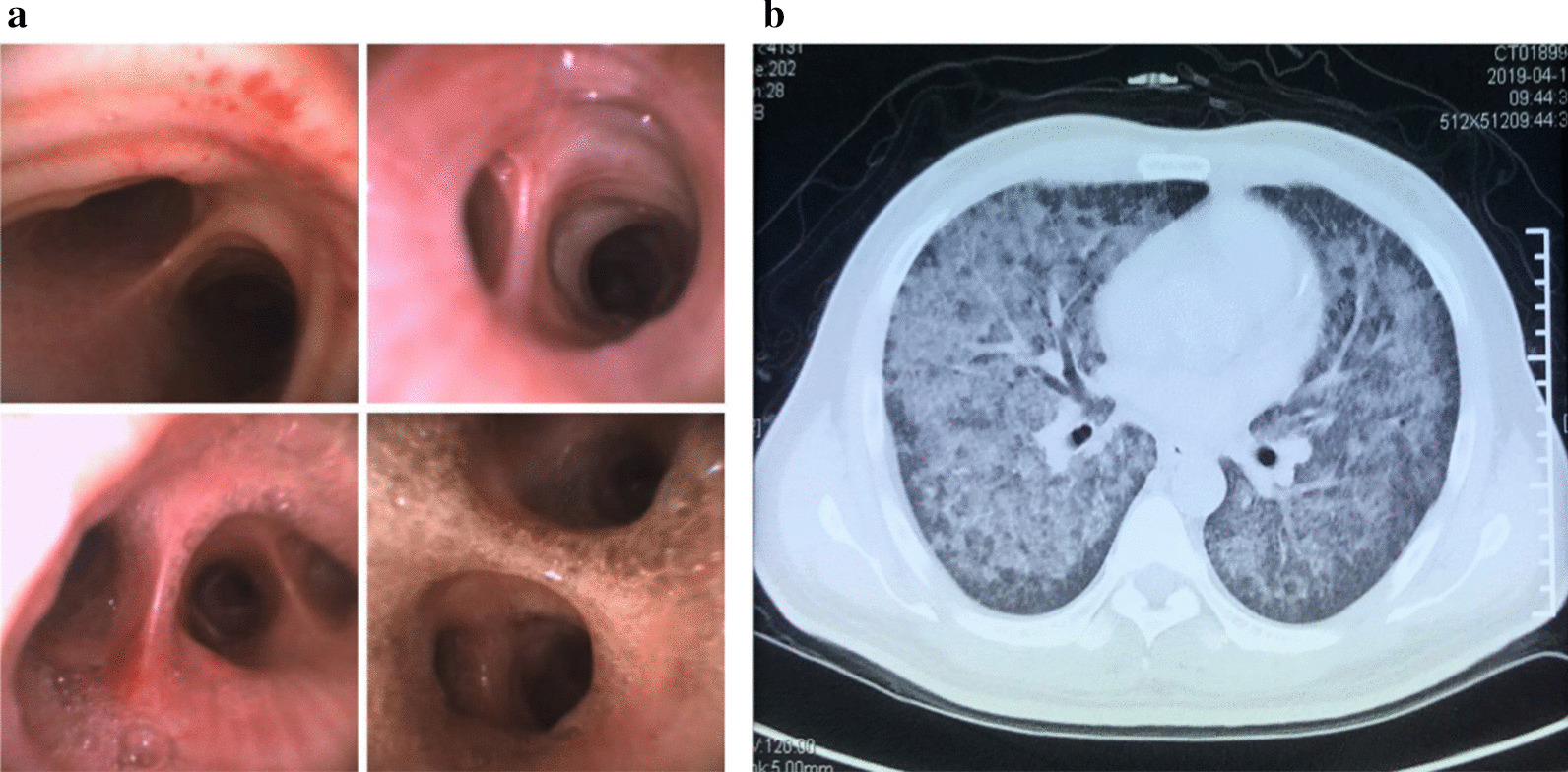
Bronchoscopy and chest computed tomography image. **a**. Images taken during bronchoscopy showing miliary nodules. **b**. Chest computed tomography image showing ground-glass opacifications in both lungs

A 44-year-old male patient (Han Chinese ethnicity) experienced dry cough, chest tightness, and shortness of breath for 2 weeks and was transferred to the emergency department at Shuguang Hospital. The patient has been previously diagnosed with type 2 diabetes and hepatitis C and had a history of intravenous drug use. Upon admission, we conducted a physical examination that showed a body temperature of 39.3 °C, pulse of 117 beats per minute, respiratory rate of 22–30 breaths per minute, and blood pressure of 120/84 mmHg. The patient had normal development and body shape, and was conscious but showed signs of malnutrition. Skin was normal without yellow dye, erythema, rash, or pigmentation. Lymph glands were not enlarged. Chest auscultation indicated rough breath sounds and moist rales in both lungs. Furthermore, bronchoscopy and chest computed tomography (CT) scan revealed miliary nodules and ground-glass opacifications in both lungs (Fig. 1a, b).

Next, we carried out routine laboratory tests, and the results were as follows: SpO_2_ 90% (breath via facial mask, FiO2 50%), PaO_2_ 52 mmHg, PaCO_2_ 26 mmHg, pH 7.5, Base Excess (BE) 0.6 mmol/L, Lactate concentration (Lac) 2.1 mmol/L, Oxygenation Index (OI) 101 mmHg, white blood cell count (WBC) 7.3 × 10^9^/L, Neutrophil (N) 89%, C-reactive protein (CRP) 111.7 mg/L, procalcitonin (PCT) 0.67 ng/ml, total bilirubin (TBiL) 31.8 μmol/L, alanine aminotransferase (ALT) 59 U/L, aspartate aminotransferase (AST) 135 U/L, albumin (Alb) 24 g/L, and CD4+/CD8+ 0.7. The microbiological culture of sputum reported Gram-positive and Gram-negative cocci and *Candida albicans*. Acid-fast stain, *Mycoplasma pneumoniae* tests were negative. Bacterial and fungal culture of bronchoalveolar lavage fluid (BALF) were negative. Galactomannan test (GM test) using BALF was negative. Blood culture was negative. Serum β-d-Glucan test (G test) was negative. Tuberculosis immunoglobulin M (IgM) and immunoglobulin G (IgG) tests were negative. However, the interferon alpha release assays including T-SPOT.TB and QuantiFERON-TB Gold (QFT-G) were positive. As a result, we performed metagenomic next-generation sequencing (mNGS) tests on BALF (DNA sequencing), which reported one read of *Mycobacterium tuberculosis* and 43 reads of *Tropheryma whipplei*. To further confirm the presence of MTB, we carried out MTB fluorescent polymerase chain reaction (PCR) test, which was positive for MTB with no detection of rifampicin and isoniazid antibiotic resistance (Table 1).

**Table 1.** Results of MTB fluorescent PCR

Test	Molecular target	Result
MTB	*rpoB* gene	Positive
Rifampicin resistance	*rpoB* AA 507-512 mutation	Negative
	*rpoB* AA 512-520 mutation	Negative
	*rpoB* AA 520-528 mutation	Negative
	*rpoB* AA 528-533 mutation	Negative
Isoniazid resistance	*katG* (315 G>C)	GG
	*katG* (−15 C>T)	CC

MTB: Mycobacterium Tuberculosis, AA: amino acid

Combining clinical manifestation and above-mentioned microbiological data, we diagnosed the patient with community-acquired pneumonia with acute respiratory distress syndrome (ARDS) that was caused by coinfection of MTB and TW. We then performed endotracheal intubation and mechanical ventilation and administered trimethoprim/sulfamethoxazole (TMP/SMX) 1.44 g three times per day orally, isoniazid 0.3 g once per day orally, rifapentine 0.6 g twice per week orally, amikacin 0.6 once per day intravenous guttae (ivgtt), and ethambutol 0.75 g once per day orally.

**Fig. 2:**
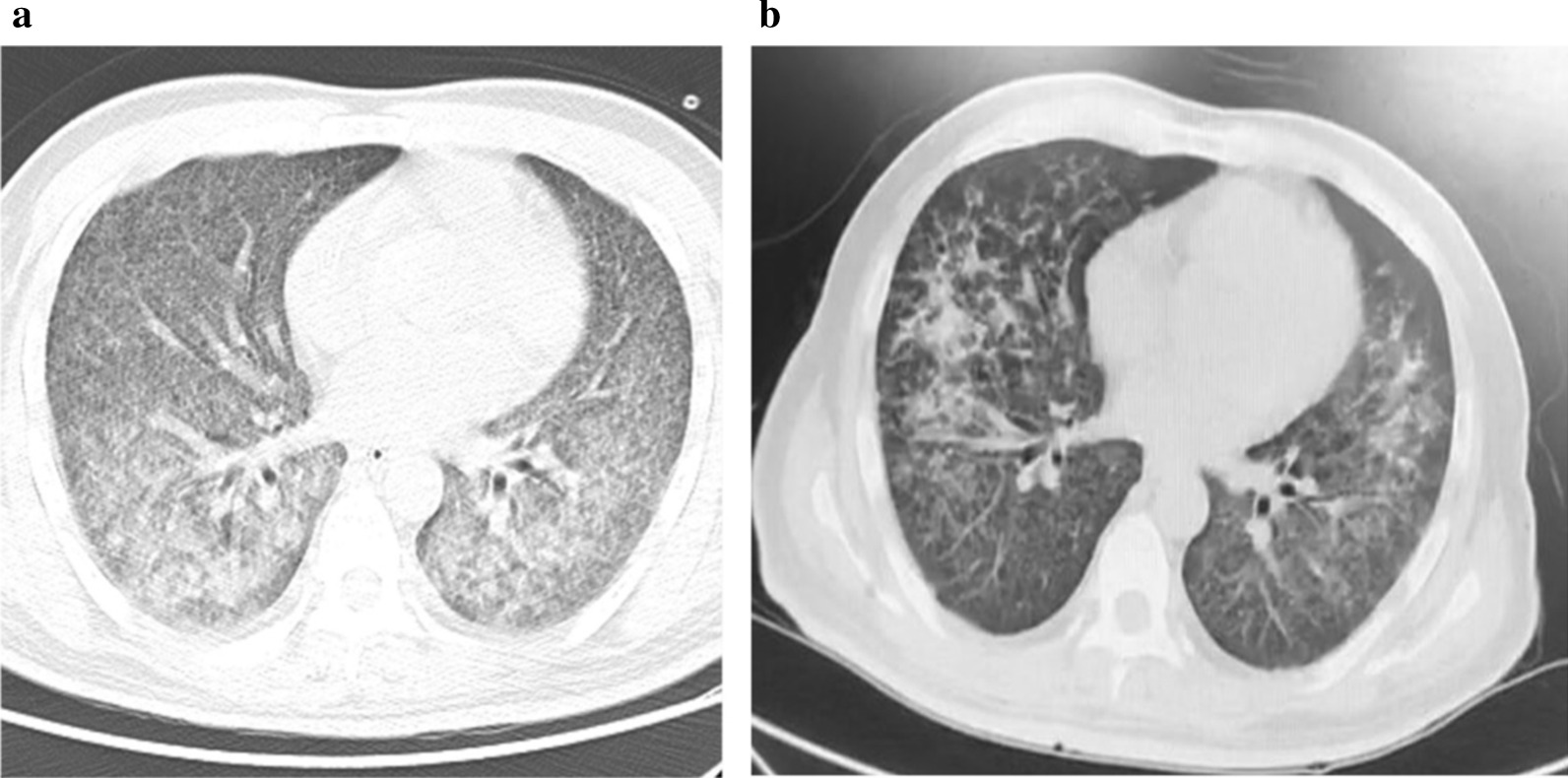
Chest computed tomography images after treatment. **a**. Chest computed tomography after 14 days of treatment. **b**. Chest computed tomography after 21 days of treatment

After 14 days of treatment, the patient’s body temperature returned to normal with improvement of blood oxygen saturation. Chest CT scan displayed absorption of ground-glass opacifications (Fig. 2a). Ventilator was removed, and the patient was given high-flow nasal cannula. At 21 days posttreatment, chest CT scan showed significant absorption of miliary nodules (Fig. 2b).

## Discussion and conclusions

Tuberculosis may lead to severe sepsis, which can be fatal in patients with immunodeficiency, including patients with HIV, cancer, and immunological disorders [5]. TW could cause both acute and chronic infections and can colonize healthy individuals. On the other hand, TW is known to cause acute pneumonia, which was previously reported to be associated with aspiration pneumonia as well as acute respiratory distress syndrome (ARDS) [6]. TW infection may initially lead to fever and idiopathic or migratory arthritis and progress to cause steatorrhea, weight loss, hepatosplenomegaly, and ascites. Notably, approximately 30% of TW infection affects the lung, resulting in respiratory symptoms including dry cough, chest pain, shortness of breath, and pleural adhesion. There are at least 11 clinical reports involving TW and interstitial lung disease [7]. In a study using mNGS and BALF samples collected from 210 intensive care unit (ICU) patients with pneumonia, six (3%) contained sequencing reads specifically aligned to TW [8]. In addition, TW is commonly found in HIV patients, who have poses a higher risk of pulmonary infection [9]. Considering that pulmonary alveoli contain macrophages, which TW mainly targets, they may serve as primary sites of TW colonization or infection.

Diagnosis of TW is usually difficult owing to the paucity of microbiological tests. The antibody titer is low in serum, making a serology test ineffective at diagnosing TW infection [10]. Periodic acid–Schiff (PAS) stain has been a traditional and primary diagnostic method for TW. However, the presence of other pathogens including *Pneumocystis jirovecii* and *Mycobacterium avium* may produce false-positive results [11]. Moreover, TW is auxotrophic and takes ~18 hours for one proliferation cycle, making bacterial culture impractical. However, with the development of nucleic acid tests, PCR-based and sequencing-based molecular assays have proven to be effective for TW detection and, hence, have become the main diagnostic choice [12].

Guidelines for treating TW infection are currently lacking. Literature suggests a variety of antimicrobial regimen, including penicillin 12,000 units/day [13], tetracycline 600 mg/day [14], TMP/SMX 160/800 mg/day [15], TMP/SMX 160/800 mg/day with ceftriaxone 2 g/day or meropenem 3 g/day [16], and doxycycline 200 mg/day with hydroxychloroquine 600 mg/day [15]. In our case, the patient has type 2 diabetes and has been serving a sentence in prison. According to one study, the incidence rate of MTB infection in prison (657/100,000) is 3.75-fold higher than in the general population [17]. The malnutrition state, positive for blood T-SPOT.TB and QFT-G tests, as well as miliary nodules seen on chest CT were all consistent with tuberculosis. The diagnosis was further corroborated by mNGS and PCR. The low read number may be due to difficulties in nucleic acid extraction, as the cell walls of MTB are rich in lipid and hard to break by either chemical or physical means. In addition to miliary nodules, ground-glass opacifications were also evident on chest CT that indicate infection of additional pathogens. The ratio of CD4+/CD8+ was less than 1, suggesting an immunodeficient state of the patient. We initially did not administer anti-TB treatment but were able to observe some absorption of exudation around miliary nodules after use of antibiotics. By adding anti-TB therapy, most pulmonary lesions were absorbed within 3 weeks (Fig. 2b). Moreover, we ruled out pulmonary alveolar hemorrhage and proteinosis because of the following evidence: (1) blood was not present in BALF; (2) the patient has been diagnosed with hepatitis C, which was classified as Child–Pugh class A with normal international normalized ratio (INR); (3) BALF was clear, and no precipitates were seen after centrifugation; and (4) PAS stain was negative. As a result, combining these clinical observations with presence of MTB and TW sequencing reads by mNGS, we concluded that the patient had a pulmonary coinfection of MTB and TW.

In summary, TW is less commonly detected in the general population than in immunocompromised individuals. Opportunistic infection and coinfection of TW is possible. Pulmonary TW infection is usually indistinguishable from other bacterial infections. As a result, nucleic acid tests such as mNGS are especially valuable for identification and differential diagnosis of TW infection. We also confirmed in our case that oral administration of TMP/SMX was effective in treating pulmonary TW infection.

## Data Availability

All pertinent data are presented in this case report.

## Abbreviations

mNGS: Metagenomic next-generation sequencing
MTB: *Mycobacterium tuberculosis*
TW: *Tropheryma whipplei*
ARDS: Acute respiratory distress syndrome
TMP/SMX: Trimethoprim/sulfamethoxazole
BALF: Bronchoalveolar lavage fluid
